# Behavioral Intervention to Prevent HIV/AIDS Among Young Adults Using Motivational Culture-Based Communication: Protocol for a Research and Development Study

**DOI:** 10.2196/72996

**Published:** 2025-11-28

**Authors:** Moh Khotibul Umam, Agus Setiawan, Henny Permatasari, Muchtaruddin Mansyur

**Affiliations:** 1 Nursing Department, Faculty of Health Sciences, Universitas Pekalongan, Sriwijaya Street 03, Pekalongan, Central Java, 51111, Indonesia, 62 81229181869; 2 Department of Community Nursing, Faculty of Nursing, University of Indonesia, Depok, West Java, Indonesia; 3 Department of Community Medicine, Faculty of Medicine, University of Indonesia, Depok, West Java, Indonesia

**Keywords:** HIV/AIDS prevention, community-based intervention, young adults, intervention development, cultural health messaging, motivational strategies

## Abstract

**Background:**

HIV/AIDS is an epidemic in 190 countries, with Indonesia having more than half a million people living with the virus. Risk factors include risky sexual activity and alternating needle usage. The majority of cases are young adults (aged 18 to 30 years). The Indonesian National HIV/AIDS strategy aims to reduce new cases to less than a thousand per year. Nowadays, effective prevention involves avoiding free sex and adopting healthier lifestyles.

**Objective:**

This research aimed to explore young adults’ needs, perceptions, and cultural aspects of HIV/AIDS prevention, develop the intervention model, analyze its effectiveness, and examine its effect on HIV prevention behavior.

**Methods:**

We conducted the study in three phases: qualitative research, model development, and quantitative research. In the first phase, we used descriptive phenomenology and focus group discussion to collect data from young adults and stakeholders in Batang and Pekalongan regencies. The second phase developed a motivation model for HIV/AIDS prevention using motivational culture-based communication (MK-U) through website media. We designed the model in consultation with an information technology team and experts to ensure its suitability. The third phase involved a pre- and posttest with a control group quasi-experiment involving young adults aged 20‐30 years in Batang and Pekalongan districts. The intervention group received culture-based motivational interviewing, while the control group received standard counseling. We analyzed the data using a generalized linear model and multivariate analysis of variance.

**Results:**

We expected to develop the MK-U model for HIV prevention behavior among young adults. In 2025, we implemented a community-based nursing research program for the MK-U model to prevent HIV/AIDS.

**Conclusions:**

In this research, we compare the results with similar studies, which will benefit program implementers and policymakers in both regions and enhance the effectiveness of HIV prevention programs.

## Introduction

HIV/AIDS has become an epidemic in nearly 190 countries around the world. The prevalence in the last 40 years has reached more than 80 million people worldwide [1]. The estimated number of people living with HIV/AIDS in Indonesia from 2005 to 2021 reached 543,100. The three highest risk factors for HIV/AIDS transmission in Indonesia are risky sex in heterosexuals and homosexuals, as well as the use of alternating needles among injecting drug users. The age range of 25‐49 years accounts for the majority (68.1%) of HIV/AIDS cases. This is associated with the window period of HIV/AIDS (8‐10 years), so it is likely that HIV transmission occurs in the age range of 20‐30 years. The number of HIV/AIDS cases in the young adult group also increased by approximately 1% compared to 2021 [2].

Despite a notable increase in HIV infections among young adults aged 20‐30 years in Indonesia, existing programs have not been adequately adapted to address the evolving needs of this demographic. There remains a significant lack of knowledge about HIV/AIDS and youth-friendly prevention, limiting accessibility and engagement [3-6]. Furthermore, digital and social media platforms—highly relevant for youth outreach—have been underutilized in HIV prevention [7]. Engagement efforts targeting individuals aged 15‐30 years have been insufficient, despite evidence indicating early age of transmission and risk exposure [8]. In addition, cultural and religious norms continue to impede open discussions about sexual health and the promotion of condom use, further exacerbating the challenges in HIV prevention among youth [6,9]. This trend is likely to persist, and potentially worsen, if comprehensive and targeted efforts are not implemented to curb the transmission of HIV/AIDS in Indonesia, particularly among young adults.

In the absence of an effective vaccine for HIV, prevention efforts primarily rely on behavioral strategies aimed at reducing the risk of transmission. One widely recognized framework is the ABC approach—abstinence, be faithful, and condom use—which promotes risk avoidance through sexual abstinence, monogamous relationships, and consistent condom use [10]. Behavioral change interventions (BCIs) are central to HIV prevention, as they support individuals in adopting safer practices and healthier lifestyles. Among these, community outreach and mentoring have proven to be effective strategies. Such interventions often include motivational health education to discourage casual sexual encounters, the development of problem-solving skills, and access to counseling services, all of which contribute to informed decision-making and risk reduction [11,12].

Primary prevention serves as the frontline strategy in reducing the incidence of disease, employing approaches at the individual, family, and community levels [10]. Its effectiveness is significantly enhanced when supported by strong commitment and collaboration from both the community and government. One key primary prevention strategy aimed at reducing risky sexual behavior among young adults involves delaying sexual initiation and promoting sexual abstinence. Avoiding early and unprotected sexual activity during young adulthood has been shown to significantly lower the risk of HIV/AIDS transmission.

In the provision of nursing care, it is essential for nurses to apply theoretical models that are designed to guide and support individuals, families, and groups in the prevention, management, or recovery from illness [13]. One such nursing theory applicable to group-based care is Johnson’s Behavioral System Model (JBSM). This conceptual model is founded on the premise that nursing practice should be guided by an understanding of behavioral systems, analogous to how medical practice is grounded in the biological systems of the human body. Johnson’s model emphasizes the importance of assessing and managing human behavior systematically, thereby enabling nurses to develop comprehensive care plans that promote behavioral balance and overall well-being.

To maintain balance within each behavioral subsystem, individuals must engage in three essential activities: protection, maintenance, and stimulation. When these functional requirements are met across all subsystems—namely attachment and affiliation, dependency, ingestion, elimination, sexuality, aggression, and achievement—the individual is considered to be in a state of health that supports adaptive functioning and self-preservation [14,15]. Johnson proposed four key assumptions regarding the structure and function of each behavioral subsystem: drive, set, choice, and observable behavior. These components collectively form the foundation of the JBSM, which provides a structured framework for nursing interventions. JBSM can be effectively applied in community-based nursing care, particularly in group settings, to guide the prevention of diseases such as HIV/AIDS by promoting behavioral balance and adaptive responses among at-risk populations.

The behavioral approach is recognized as an effective method for HIV/AIDS prevention, as it enables individuals to modify risky behaviors and adopt healthier lifestyles [16]. BCIs represent a commonly used strategy aimed at reducing high-risk behaviors and promoting the maintenance of positive behaviors through a series of activities tailored to the specific needs of target groups. These interventions seek to create supportive environments at both individual and collective levels. BCIs can be implemented through the delivery of communication, information, and education strategies or through structured group education programs [17,18].

BCIs can be further developed into comprehensive intervention models designed to influence individual behavior within the context of HIV/AIDS prevention [19]. This development may involve pilot testing within research studies to ensure the model’s applicability and effectiveness. The formulation of such a model may require the integration of existing theoretical frameworks, the consideration of influencing factors, and contextual adaptation based on the specific challenges identified in the study setting. One widely applied framework is the information, motivation, and behavioral skills (IMB) model, which has been used extensively to identify modifiable determinants of behavior and to guide the development of effective behavioral interventions [20,21].

However, several limitations have been identified in the IMB model. First, the information construct within the model has been shown to be a relatively weak and sometimes inconsistent predictor of behavior. While information is necessary, it alone is often insufficient to drive behavioral change. For instance, a study by Eggers et al [22] found that behavioral skills predicted actual behavior through motivation, as hypothesized by the IMB model, but not directly through knowledge about sexually transmitted infections. The second limitation concerns the overlap between the constructs of information and motivation, which are often not clearly distinct in practice. This interdependence presents challenges in testing and validating the model, as it complicates the interpretation of their individual effects. Lastly, the IMB model does not incorporate environmental and cultural factors, which are critical in predicting and explaining behavior, particularly in diverse sociocultural contexts. The exclusion of these factors limits the model’s predictive power and its applicability across different populations [23].

Therefore, in response to the limitations identified in the IMB model, a modification will be undertaken by integrating the motivation construct of the IMB model with another theoretical framework, the integrated change model (ICM). This integration aims to examine additional variables that may enhance the explanatory power of the model by accounting for human characteristics, environmental factors, temporal changes, and contemporary cultural influences. The ICM conceptualizes behavior change as a process comprising three phases: awareness, motivation, and action [24,25]. Moreover, the ICM incorporates predisposing factors, including social and cultural determinants, which address a notable gap in the IMB model that does not explicitly consider these influences. This study will utilize a culturally contextualized communication approach as a novel application of the combined model. The integrated and modified framework, termed the motivational culture-based communication (MK-U) model, will be empirically tested to assess its effectiveness in enhancing self-awareness, behavioral intentions, and preventive actions related to HIV/AIDS. This evaluation will be conducted through a series of research phases aimed at validating the model’s applicability within the target population.

One of the primary objectives of the Indonesian National HIV/AIDS Strategy is to reduce the annual incidence of new HIV infections to fewer than 1000 cases. Achieving this ambitious target necessitates a phased, multilevel approach, with particular emphasis on reaching vulnerable populations such as young adults aged 20 to 30 years. District- or city-level interventions targeting this demographic are crucial components of this strategy, as localized efforts allow for tailored prevention and treatment initiatives that reflect the unique epidemiological and sociocultural contexts of each area. However, preliminary data from two districts in Central Java—Batang and Pekalongan—indicate that current efforts remain suboptimal in effectively engaging this key age group.

Several factors contribute to this shortfall. First, epidemiological data show that the majority of new HIV/AIDS cases (65.4%) occur within the broader age range of 25‐49 years, suggesting that prevention efforts may not be sufficiently targeted or timed to intercept risk behaviors early among younger adults before infection occurs. Second, mobile voluntary counseling and testing (VCT) services, which serve as critical entry points for diagnosis and linkage to care, have predominantly focused on key populations such as sex workers and men who have sex with men, inadvertently overlooking the broader young adult population at risk. Additionally, these VCT activities have yet to fully leverage internet-based platforms and eHealth approaches, such as websites and mobile apps, which are increasingly recognized as effective tools for outreach, education, and engagement among digitally connected youth.

Furthermore, the absence of active youth organizations dedicated to HIV/AIDS awareness and advocacy limits peer-to-peer education and support mechanisms that are vital for influencing behavioral change within this age cohort. The role of the AIDS Commission (Komisi Penanggulangan AIDS [KPA]), which is pivotal in coordinating multisectoral HIV/AIDS responses at the district level, has also been constrained by budgetary restrictions exacerbated by the lingering economic impacts of the COVID-19 pandemic. This financial limitation has hindered the commission’s capacity to sustain outreach programs, implement innovative interventions, and maintain consistent community engagement.

Addressing these gaps requires a comprehensive, multifaceted approach. Integrating digital health technologies into VCT and prevention programs could enhance accessibility and appeal to young adults who are frequent users of social media and online platforms. Strengthening partnerships with youth-led organizations and incorporating peer-led models may increase trust and relevance in prevention messaging. Moreover, advocating for the restoration and expansion of funding for local AIDS commissions is critical to ensure sustained program delivery. Finally, employing data-driven targeting that accounts for local epidemiology and behavioral risk patterns can improve the precision and impact of interventions aimed at reducing new infections among young adults, ultimately supporting Indonesia’s goal of curbing the HIV epidemic.

Based on this background, this study seeks to develop a website-based outreach model targeted at young adults as a strategic intervention for HIV/AIDS prevention, with the ultimate goal of contributing to the reduction of HIV/AIDS incidence in Indonesia. The research is grounded in the urgent need for culturally sensitive, youth-oriented approaches that leverage digital platforms to promote awareness, intention, and behavior change. Accordingly, the question of this research is how effective is the MK-U model in increasing self-awareness, behavioral intention, and preventive behavior related to HIV/AIDS among young adults aged 20 to 30 years in Central Java, Indonesia.

The objectives of this research are as follows: (1) to explore the needs, perceptions, and behavioral determinants of young adults in relation to HIV/AIDS prevention, (2) to examine the programs, strategies, and sociocultural dimensions employed by local stakeholders in HIV/AIDS prevention efforts targeting young adults, (3) to design and develop the MK-U model framework, including an intervention module, workbook, evaluation instruments, and a web-based outreach platform tailored to the needs of young adults, (4) to assess the effectiveness of the MK-U model in enhancing self-awareness and behavioral intention related to HIV/AIDS prevention among young adults, and (5) to evaluate the impact of the MK-U model intervention on the adoption of preventive behaviors among young adults at risk of HIV/AIDS.

## Methods

### Study Design

**Figure 1.**
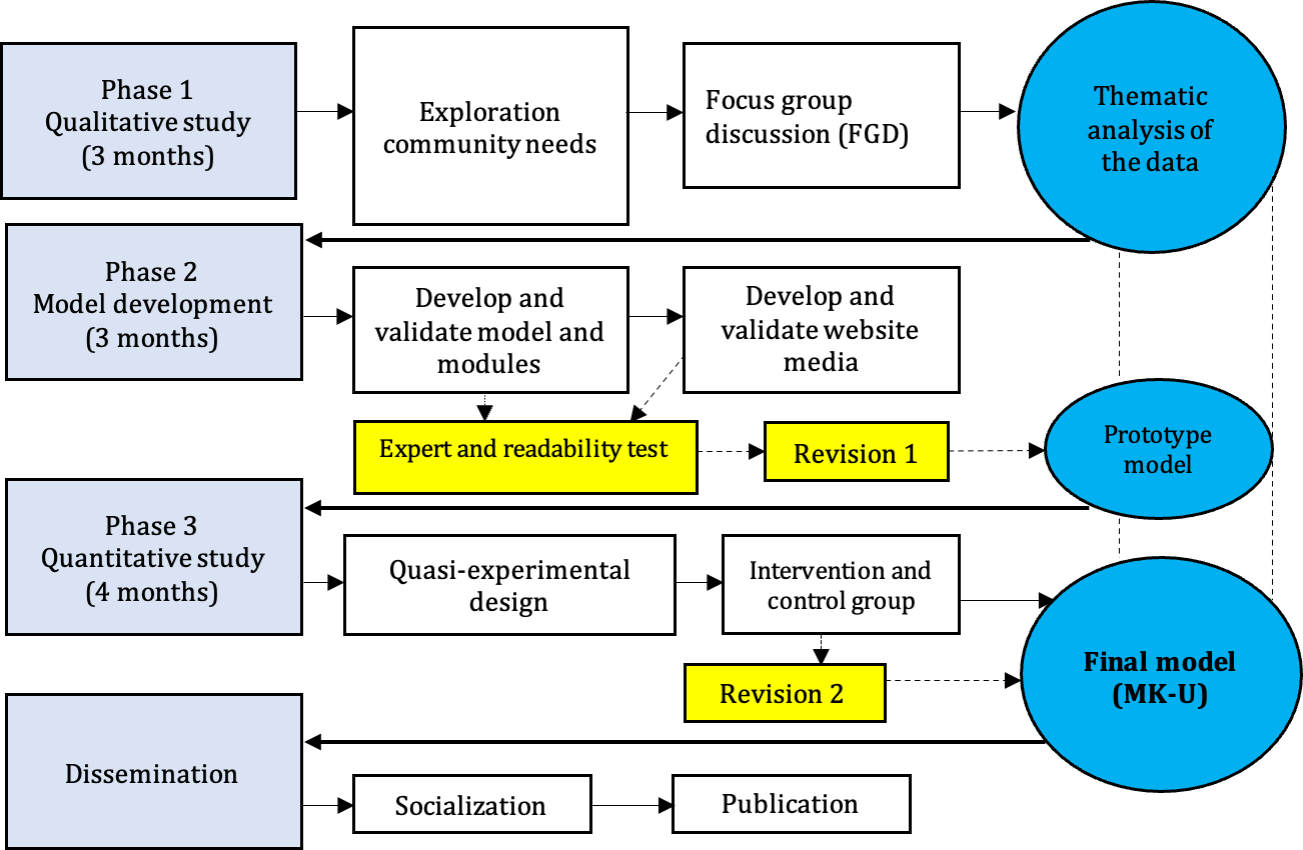
Study outline.

This study used the research and development method to develop the MK-U model. This method was used to create specific products or models and to evaluate their effectiveness. Three phases comprised the research process: qualitative research, model development, and quantitative research, in order to produce a specific product or model. The duration of phases is described in the study outline (see Figure 1).

### Phase 1: Qualitative Study

#### Study Setting

The research was conducted in a community setting in Pekalongan and Batang Regencies, Central Java, Indonesia. The primary objective of the first phase of the research explored the perceptions and needs of young adults in order to enhance their HIV/AIDS preventive practices. In addition, exploration needed to be done with stakeholders, namely nurses as program implementers, the Health Office, the District AIDS Commission, and nongovernmental organizations (NGOs), to explore cultural aspects and programs that have been carried out for HIV/AIDS prevention in Pekalongan and Batang Districts.

The research approach carried out in this study was a descriptive phenomenology with focus group discussion (FGD) as a data collection method. This approach was very effective for studying, better understanding, and describing important phenomena from human experience or participants [26,27]. The results of this study were used as material to develop the MK-U model by utilizing website media.

#### Participants

The study consisted of two distinct groups of participants: young adults and stakeholders from the Batang and Pekalongan regency areas. The young adult group consisted of a total of 20 participants, separated into two subgroups. Specifically, there were 10 male young adults and 10 female young adults from each regency. Engaging both male and female young adults in this research enabled the capture of gender-specific insights concerning knowledge gaps, communication patterns, stigma, and prevention behaviors within the key demographic at heightened risk of HIV infection. This gender-inclusive approach facilitated a more comprehensive understanding of the differential factors influencing HIV-related attitudes and practices, thereby informing the development of targeted interventions that address the unique needs and challenges faced by each gender group in the context of HIV prevention.

The stakeholder’s group consisted of a total of 20 participants as well, separated into two subgroups. Specifically, there were 10 participants from health workers and 10 participants from nonhealth workers from each regency. The health workers included the health care provider at the public health office (Dinas Kesehatan) and nurses at public health center (Puskesmas). This group consisted of 2 participants from public health office and 8 participants from public health center (Puskesmas) in each regency. This study uncovered provider-level stigma, training needs, and systemic barriers that hinder effective service delivery and coordination of HIV care. By including health workers from both administrative and frontline roles, the research facilitated a comprehensive exploration of their attitudes, knowledge levels, and existing training gaps. Understanding these dimensions was essential, as they directly influenced the accessibility, quality, and responsiveness of HIV prevention and care services at the local level. This approach also highlighted structural and institutional challenges that must be addressed to strengthen the overall effectiveness of community-based HIV interventions.

In addition, nonhealth stakeholders participating in this study included the District AIDS Commission (KPA) coordinators, NGO coordinators, and community leaders from both Batang and Pekalongan districts. This group comprised 3 participants from the KPA, 4 from NGOs, and 3 community leaders in each district. FGDs continued until data saturation was reached within each stakeholder group. The inclusion of nonhealth stakeholders was critical for capturing the broader social dynamics, cultural norms, and collaborative mechanisms that shape HIV prevention efforts and influence stigma at the community level. Their perspectives provided valuable insights into how societal attitudes, community leadership, and local advocacy shape the effectiveness of existing interventions. Furthermore, this approach allowed for a deeper understanding of the strengths and limitations of current partnerships among governmental, nongovernmental, and community actors in addressing HIV-related challenges.

#### Participants Recruitment

The research used purposive sampling as the sample technique. The participants focused on the specified inclusion requirements, which require them to be young people between the ages of 20 and 30 years, unmarried, and eager to actively participate. The exclusion criteria pertained to young individuals who have mental illnesses. NGOs were engaged to facilitate the recruitment of key informants in both areas. Meanwhile, stakeholder participants were invited through agency leaders to represent them in the research process.

#### Data Collection

The researcher recruited a research team consisting of four people to assist in the research process. Semistructured interview guidelines were used to perform FGDs. Each FGD group had a session that lasted roughly 90‐120 minutes. Throughout the FGD process, the research team transcribed and recorded participants’ names and comments. The seating arrangement was based on the attendees’ preferences. Researchers recorded the whole FGD session, capturing the process from the beginning to the end, using a voice recorder and digital camera. If one of the informants was absent, the researcher confirmed the reason for not being able to attend and then recorded it in the field notes. The FGD process proceeded based on the number of participants present. The research team summarized and analyzed each FGD session, then presented a written or oral report to the participants. All comments and grammatical mistakes were reviewed and fixed as necessary.

#### Data Analysis Plan

A content and thematic analysis was used for data analysis using four steps, including bracketing, analyzing, intuiting, and describing [26,28]. Then, the credibility technique was used to explain the degree or truth value of the data generated, including the data analysis process from the research conducted. We implemented two strategies to ensure data validity: the first involved the researcher, who also used FGD discussions and participant observation to ensure the data were truly accurate. The second strategy was member check, which involved anyone who participated in the research in checking the findings to ensure that they were consistent with their experience. We also used descriptive validity and interpretative validity techniques to ensure the validity of the data. Descriptive validity refers to the report’s factual accuracy, whereas interpretive validity refers to the report’s interpretation. The report was based on the participants’ language and relied as much as possible on their own words and concepts. When summarizing the results, the researcher was cautious because there were jumps from the participants’ words and actions that affected the conclusions. The entire FGD process was in Bahasa Indonesia. All data were performed manually with Microsoft Excel, ensuring a thorough and reflective engagement with the data.

### Phase 2: Model Development

#### Study Procedures

The goal of phase II research was to develop a motivation model for HIV/AIDS prevention among young adults using MK-U through internet media. After obtaining findings from the first phase of the research, researchers proceeded with reviewing literature derived from textbooks and previous articles published in scientific journals. This literature review focused on the examination of HIV/AIDS preventative behavior among young adult populations, using a cultural framework facilitated by online media or telehealth platforms. Thus, the present study ascertained and established the development of this model by analyzing the findings of phase 1 research and three conceptual models, namely the IMB model [21], the ICM [24], and the JBSM [29], which were used in this study.

Subsequently, the researchers collaborated with an information technology (IT) team to design a website that aligns with the objectives. Following this, the researchers and the IT team scrutinized the website’s requirements, pinpointing the system’s users and defining its functional and nonfunctional needs, in order to offer a preliminary outline of the system’s construction during its implementation. Researchers also formed a content creation team to create website materials and media tailored to user needs. Finally, using reference sources that aligned with the topic and needs, researchers formed a team to prepare a draft module that integrates with the MK-U model. We then subjected the draft modules and workbooks to expert and readability tests to ensure their suitability for use.

#### Expert and Readability Tests

We conducted the first expert consultation to gather input, suggestions, and approval for the draft website, module, and workbook. Then, we compiled it before the trial. The instrument for the expert consultation was a questionnaire. The researcher presented a series of statements and asked the expert to clearly assess their appropriateness for the young adult age group. We used these statements as content on the website, developed modules, and sourced workbooks from several relevant pieces of literature. Regarding the website draft, it was consulted with technology experts who were experienced in making websites. Experts in content creation also provided advice on incorporating content like videos and photos into the website.

Then, specifically for the nursing training module, an expert test was conducted to determine the training materials for counseling with a culturally based motivational interviewing (MI) approach or communication style for health care nurses. In the context of initial content validation for a small-scale training module, the engagement of three to five subject-matter experts is widely regarded as methodologically sound and appropriate. This range is supported in the literature as sufficient for obtaining expert judgment on the relevance, clarity, and importance of instructional materials, particularly during early stages of development [30].

Following the initial identification of key materials, a panel of experts was invited to review the preliminary list to evaluate its completeness and relevance. Experts were encouraged to suggest additional materials they deemed important but were not included in the original compilation. Based on their feedback, the list was revised accordingly. Subsequently, a second round of expert review was conducted, during which the experts were asked to rank each material on a scale from 1 to 4, with higher scores indicating greater importance for inclusion in the training module. This ranking process aimed to establish a prioritized understanding of content relevance, ensuring that the most critical materials would be emphasized in the final training design.

As part of the expert validation process for the training module, a 1‐4 ranking scale will be employed to assess the relative importance of selected content materials. Ranking is particularly effective in expert-based content validation as it requires participants to differentiate and prioritize items, thereby offering clearer insights into which materials are perceived as most essential. Unlike rating scales, ranking forces a comparative judgment across items, minimizing the risk of score inflation or central tendency bias. The use of a 4-point ranking scale is both purposeful and practical. Given the small number of items under consideration, a limited set of ranking options helps maintain cognitive ease for the experts while still producing meaningful distinctions between materials. Additionally, the even-numbered format avoids the inclusion of a neutral midpoint, thereby encouraging more decisive judgments regarding each item’s relative importance. This enhances the discriminative power of the expert feedback and supports better prioritization in the final training content. The purposive sampling technique will select experts based on the inclusion criteria, which include panels and experts actively involved in HIV/AIDS prevention efforts at both national and regional levels for a minimum of 5‐10 years. Additionally, we included technology experts who have accumulated at least 5‐10 years of experience in IT and eHealth website development.

A pilot study was conducted to evaluate the usability, functionality, and content effectiveness of the developed website or training material prior to full-scale implementation. A purposive sample of 5 to 10 participants representative of the target audience was recruited, as this size was generally sufficient to identify most usability issues. During the pilot, participants were asked to complete specific tasks while navigating the website or materials, allowing the researcher to observe ease of navigation, clarity of content, technical performance, and overall user satisfaction. Data collection included observation checklists, standardized usability questionnaires, and qualitative feedback obtained through interviews. Quantitative data were analyzed descriptively to assess usability ratings, while qualitative data were thematically analyzed to identify common barriers and areas for improvement. Findings from the pilot study informed necessary revisions to optimize the user experience and enhance the effectiveness of the training content before the main implementation phase.

### Phase 3: A Quasi-Experimental Study

#### Study Design

After the draft model was developed in the second stage of research, the next stage of research was to test the effectiveness of the model. In research and development, we used quantitative research methods to test the model’s effectiveness. The research design in this study was a pre-post test with a control group quasi-experiment design.

#### Study Population and Eligibility Criteria

Young adults aged 20‐30 years, residing in Batang and Pekalongan districts, possessing internet-connected gadgets, and not married met the inclusion criteria. Young adults with a history of mental disorders and HIV/AIDS infection met the exclusion criteria.

#### Sample Size

The formula’s sample calculation indicates that this study included 109 samples for both the intervention and control groups. To anticipate dropout, the sample was increased by 20% [31]. Therefore, the total sample in this study was 130, consisting of 65 for the intervention group and 65 for the control group.

#### Recruitment

We utilized simple random sampling as a sampling technique, adhering to the following sequences: (1) prospective respondents will register as members on the provided website; (2) after becoming members, they will be asked to indicate their willingness to participate in the study; (3) respondents who meet the inclusion and exclusion criteria will be randomly selected to participate in the research process; and (4) respondents may refuse participation or withdraw at any time without consequence. To ensure allocation concealment, the randomization sequence was generated by an independent researcher using computer software, and assignments were kept confidential until the point of selection to prevent selection bias. Due to the nature of the study, blinding of participants and researchers may not be feasible; however, outcome assessors were blinded to participants’ group assignments to minimize detection bias and enhance the study’s internal validity.

#### Data Collection Methods

Researchers obtained research permits from the governments of Pekalongan and Batang Regency to conduct research in these areas. The study spanned 4 months, with the research secretariat based in Pekalongan Regency and the University of Pekalongan managing the website server. Researchers made sure that the website, media content, and MK-U model materials used in the research process are suitable for use in accordance with the procedure, based on the findings from the first and second stages of research. Researchers recruited 20 public health nurses who served as counselors during the research process: 10 nurses became control group counselors, and 10 nurses became intervention group counselors. For 3 days, nurses who served as counselors in the intervention group received standardized training on the culture-based communication model and MI techniques, training in one type of MK-U model intervention, specifically counseling with a culture-based MI approach. The training included workshops, role-playing exercises, and practice sessions with feedback. A manualized intervention protocol was also developed to ensure consistent delivery across all sessions and counselors. Researchers prepared research tools such as questionnaires, modules, workbooks, training modules, and counseling process forms. Researchers prioritized respondents from the Batang and Pekalongan Regency areas who access the study's website. Researchers and their team conducted research activities with respondents in 13 weeks for the intervention and control group (see Table 1).

**Table 1.** Activities of respondents during the research process.

Weeks	Intervention group activities	Control group activities
Week 1 (Drive)	Respondents fill out the pretest. Respondents access a website that contains content and materials about HIV/AIDS. The nurse provided one counseling session with a culture-based MI approach to increase encouragement.	Respondents fill out the pretest. Respondents access a website that contains content and materials about HIV/AIDS. The nurse provided one counseling session as usual.
Week 2 (Set)	Respondents access a website that contains content and materials about HIV/AIDS. The nurse provides one counseling session with a culture-based MI approach to be able to set a change plan.	Respondents access a website that contains content and materials about HIV/AIDS. The nurse provided one counseling session as usual.
Week 3 (Choice)	Respondents access a website that contains content and materials about HIV/AIDS. The nurse provided one counseling session with a culture-based MI approach to improve the ability to choose.	Respondents access a website that contains content and materials about HIV/AIDS. The nurse provided one counseling session as usual.
Week 4 (Behavior)	Respondents access a website that contains content and materials about HIV/AIDS. The nurse provided one counseling session with a culture-based MI approach to establish positive behavior.	Respondents access a website that contains content and materials about HIV/AIDS. The nurse provided one counseling session as usual.
Week 5	Respondents access a website that contains content and materials about HIV/AIDS. The nurse provides one counseling session with a culture-based MI approach to create an action plan for HIV/AIDS prevention behavior. Respondents fill out the first posttest.	Respondents access a website that contains content and materials about HIV/AIDS. The nurse provided one counseling session as usual. Respondents fill out the first posttest.
Week 6‐8	Allocate time if a process remains unfinished.	Allocate time if a process remains unfinished.
Week 9‐12	Respondents perform activities independently.	Respondents perform activities independently.
Week 13	Respondents in the intervention group complete the second posttest.	Respondents in the control group complete the second posttest.

#### Outcomes

The intervention group conducted a pretest before initiating the MK-U model intervention action, followed by two posttests: one after the intervention was completed and another 3 months later. The intervention group received the MK-U model intervention, which included access to HIV/AIDS information on the website, modules, and a counseling process using a culture-based MI communication approach by nurses. To ensure consistency in the delivery of MI, we incorporated a fidelity monitoring process using structured observation checklists based on the Motivational Interviewing Treatment Integrity coding system. Sessions were recorded (with participant consent), and a random sample was reviewed by an independent expert to assess adherence to MI principles. Conversely, the control group experiences the standard counseling process from nurses. The objective is to ascertain the variation in the average of variables among the participants before and after the implementation of the intervention program.

#### Data Analysis Plan

The obtained data were processed using statistical software compatible with the Windows operating system. We used a generalized linear model with multivariate analysis of variance to analyze the effect of the MK-U model intervention. The analysis assumed that the dependent variables are multivariate normally distributed and that variance–covariance matrices are homogeneous across groups. To assess these assumptions, we conducted the Shapiro-Wilk test for normality, Box’s M test for equality of covariance matrices, and Levene’s test for homogeneity of variances. Independence of observations and linearity between dependent variables and covariates were also evaluated. Multicollinearity among dependent variables was assessed through correlation matrices, and significant outliers were identified using Mahalanobis distance. If any assumptions were violated, appropriate data transformations or alternative nonparametric methods were employed to ensure the validity of the analysis.

Results from the generalized linear model with multivariate analysis of variance were reported with test statistics, degrees of freedom, *P* values, and effect sizes for each dependent variable. Multivariate test results were presented to indicate the overall effect of the MK-U model intervention across outcome measures. When significant multivariate effects were detected, follow-up univariate analyses were conducted to determine which dependent variables contributed to the effect. Confidence intervals were reported alongside point estimates to indicate precision. All assumption checks, data transformations, or alternative analyses performed were transparently documented. The interpretation of results considered both statistical significance and practical relevance by examining the magnitude and direction of observed effects within the context of the study.

### Ethical Considerations

This research was conducted by considering the principles of research ethics, including consent, confidentiality, and anonymity, as well as protection from discomfort and harm [27,30]. This study has received ethical approval from the Research Ethics Committee, Faculty of Nursing of Universitas Indonesia (approval number: KET-001/UN2.F12.D1.2.1/PPM.00.02/2024, with an amendment on January 2, 2024).

To obtain consent, the researcher provided information sheets and explained research procedures to respondents. Then the participants filled out and signed the consent form to take part in the research. Participants have the right not to participate in the study. The study does not pose any harmful side effects to participants. Although no compensation is provided in the form of payment, each participant will receive a souvenir as a gesture of appreciation for completing the study procedures. Researchers maintained the confidentiality of all data collected from respondents, including personal information such as name, address, and other personal details. Meanwhile, protection from discomfort and harm referred to the researchers’ measures to ensure that participants are not in danger, are free from harmful actions, both oral and written, and are provided with comfort throughout the research process. The study also did not involve any invasive measures, nursing, or medical treatments. If there is any discomfort or disadvantage during the study, respondents can withdraw from the research process.

A password-protected computer stored data in the form of soft files, and the researcher’s office housed all research files for a period of 3 years. Upon completion of the research activities, we destroyed all documents, information sheets, and consent forms, including those stored on the computer during that period. The research results were presented in the form of data groups within the research report.

## Results

The results of this study provided evidence on the feasibility, usability, and preliminary efficacy of the MK-U web-based outreach model in promoting HIV/AIDS preventive behaviors among young adults in the districts of Pekalongan and Batang.

Quantitative data included pre- and postintervention measures of HIV knowledge, self-awareness, behavioral intention, and HIV prevention behavior. The findings are expected to inform the development of scalable, digitally enabled interventions for use by public health nurses and HIV prevention practitioners across similar contexts. This study is expected to develop an adapted, web-based outreach strategy aimed at enhancing self-awareness, behavioral intention, and HIV prevention behaviors among young adults.

This research is currently in process at Phase 1 data analysis.

## Discussion

### Principal Findings

Recent evidence underscores that the provision of information alone is insufficient to produce sustained changes in HIV prevention behaviors among young adults. While traditional interventions have often prioritized knowledge dissemination, such approaches frequently neglect the internal cognitive and motivational mechanisms that underpin behavior change. Research increasingly highlights the critical role of factors such as self-awareness, intention, self-efficacy, and behavioral skills in fostering effective and long-term preventive behaviors [32-35]. For instance, digital HIV interventions that integrate MI, behavioral theory, and user-centered design have shown greater success in promoting consistent condom use, HIV testing uptake, and preexposure prophylaxis adherence [36-39]. Moreover, engagement with these interventions has been found to be significantly mediated by digital literacy, further emphasizing the importance of tailoring approaches to users’ technological competencies [40,41]. The MK-U model directly addresses these gaps by incorporating cognitive and motivational components into a culturally contextualized, digitally enabled framework, offering a more comprehensive and responsive strategy for influencing HIV-related behaviors among young adults.

This study has several methodological and conceptual strengths. The use of a mixed-methods design enhances both the breadth and depth of the analysis. Furthermore, the study’s focus on digital literacy as both an outcome and a mechanism of change is particularly timely and relevant in the current technological context. However, certain limitations should be acknowledged. Due to the nature of the intervention, blinding of participants and implementers is not feasible, which may introduce bias in self-reported outcomes. Additionally, the study is context-specific and conducted in Bahasa Indonesia, which may limit the generalizability of findings to other cultural or linguistic populations.

### Dissemination Plan

The findings of this study are disseminated through national and international academic journals and conferences, particularly those focused on public health nursing and HIV/AIDS. It is anticipated that public health nurses will be able to utilize the resulting intervention model within their respective practice settings to enhance community-level HIV/AIDS prevention efforts.

### Conclusions

We believe that the results of the study will help provide solutions to prevent the spread of HIV/AIDS, especially among young adults in Pekalongan and Batang. We expect this research to enhance the outcomes of previous studies. The study’s findings are reported by comparing and discussing them with the results of previous similar studies. As a result, HIV/AIDS prevention program implementers and policymakers in both regions can benefit.

## Supplementary material

10.2196/72996 Checklist 1 SPIRIT checklist.

## Abbreviations

BCI: behavioral change intervention
FGD: focus group discussion
ICM: integrated change model
IMB: information, motivation, and behavioral skills
JBSM: Johnson’s Behavioral System Model
KPA: Komisi Penanggulangan AIDS
MK-U: motivational culture-based communication
SPIRIT: Standard Protocol Items: Recommendations for Interventional Trials
VCT: voluntary counseling and testing
